# Understanding uptake of COVID-19 testing, vaccination, and boosters among Spanish-speaking Latines in the United States: Qualitative insights from Spanish speakers and key informants

**DOI:** 10.1371/journal.pone.0296812

**Published:** 2024-03-07

**Authors:** Amanda E. Tanner, Mark A. Hall, Sandy K. Aguilar-Palma, Lilli Mann-Jackson, Jorge Alonzo, Alain G. Bertoni, Thomas P. McCoy, Manuel Garcia, Ana D. Sucaldito, Mari Jo Turner, Jose Robles Arvizu, Laurie P. Russell, Scott D. Rhodes

**Affiliations:** 1 Department of Public Health Education, University of North Carolina Greensboro, Greensboro, NC, United States of America; 2 Department of Social Sciences and Health Policy, Wake Forest University School of Medicine, Winston-Salem, NC, United States of America; 3 Wake Forest University School of Law, Winston-Salem, NC, United States of America; 4 Hispanic League, Inc, Winston-Salem, NC, United States of America; 5 Wake Forest Clinical and Translational Sciences Institute Program in Community-Engaged Research, Winston-Salem, NC, United States of America; 6 Division of Public Health Sciences, Wake Forest University School of Medicine, Winston-Salem, NC, United States of America; 7 School of Nursing, University of North Carolina Greensboro, Greensboro, NC, United States of America; 8 Department of Biostatistics and Data Science, Wake Forest University School of Medicine, Winston-Salem, NC, United States of America; National Institute of Public Health: Instituto Nacional de Salud Publica, MEXICO

## Abstract

**Background:**

Latine communities in the United States have been disproportionately affected by COVID-19. It is critical to gain a better understanding of the sociocultural determinants that challenge and facilitate COVID-19 testing, vaccination, and booster uptake within these vulnerable communities to inform culturally congruent strategies and interventions.

**Methods:**

In summer 2022, our community-based participatory research partnership conducted 30 key informant interviews and 7 focus groups with 64 Spanish-speaking Latine participants in North Carolina. Interviewees consisted of representatives from health and service organizations, most of whom were engaged with direct service to Spanish speakers. Interviews were conducted in either English or Spanish, depending on the preference of the participant; all focus groups were conducted in Spanish. Interviews and focus groups were conducted in person or by videoconference.

**Results:**

Twenty themes emerged that we organize into four domains: general perceptions about COVID-19; barriers to COVID-19 testing, vaccination, and booster uptake; facilitators to COVID-19 testing, vaccination, and booster uptake; and recommendations to promote testing, vaccination, and booster uptake.

**Discussion:**

Results underscore important sociocultural determinants of ongoing COVID-19 testing, vaccination, and booster uptake to consider in developing interventions for Spanish-speaking Latines in the United States. Based on this formative work, our partnership developed *Nuestra Comunidad Saludable* (*Our Healthy Community*). We are implementing the intervention to test whether trained peer navigators can increase COVID-19 testing, vaccination, and booster uptake among Spanish-speaking Latines through blending in-person interactions and mHealth (mobile health) strategies using social media.

## Introduction

In the United States, Latine communities bear a disproportionate COVID-19 burden [1–3]. As of October 2022, Latine persons comprised 25% of COVID-19 cases, despite being only 18.5% of the U.S. population (note: the term “Latine” uses a gender-neutral “e”, which replaces the gendered endings “a” and “o” as in “Latina” and “Latino” and is similar to “Latinx” and is more pronounceable when pluralized. This term is increasingly used within Latine communities) [4]. Further, Latine persons were 2 times more likely to contract, 2.8 times more likely to be hospitalized, and 2.3 times more likely to die from COVID-19 than their non-Latine White counterparts [5]. These disparities are even more acute among Spanish-speaking Latine persons in the United States [2, 5–7].

Although initial disparities have been reduced, ongoing testing, vaccination, and booster uptake remain essential to effective control of the pandemic, and complex factors affect service utilization within Latine communities. These factors include poverty, limited resources, and institutional and structural barriers. Latines may lack access to culturally congruent information about testing, vaccination, and boosters, including available services, eligibility, how to access them, and cost; be misinformed about COVID-19 risk; worry about missing work; and be concerned about engaging with government systems (including public health) [2, 7–10]. These barriers are greater among those who do not speak English and/or are undocumented [2, 6–9]. Our community-based participatory research (CBPR) partnership’s preliminary research conducted at the pandemic’s onset identified unique barriers, including: misinformation communicated through Spanish-language media/social networks and distrust in tests and vaccines (e.g., counterfeit vaccines in Latin America [6, 11–13].

A profound need remains to understand the sociocultural determinants of COVID-19 testing, vaccination, and booster uptake within Spanish-speaking Latine communities [1, 2, 6, 7, 14–16]. The heterogeneity of the Latine population is well established [17–20], and Spanish speakers experience different barriers from other Latine persons due to more pronounced economic challenges, lower acculturation, and heightened fear of government systems. Furthermore, many Spanish-speaking Latines in the United States are undocumented and/or living in mixed immigration-status households [19, 21, 22], and thus, they face unique challenges to testing and vaccination.

We qualitatively explored the sociocultural determinants of COVID-19 testing, vaccination, and booster uptake among Spanish-speaking Latines to identify intervention approaches to increase uptake of these services.

## Methods

### Community-based participatory research

Complex health challenges, including disparities in COVID-19 morbidity and mortality, can benefit from community-academic partnerships that facilitate the sharing of expertise. CBPR can enhance the quality, relevance, interpretation, and subsequent use of data and ensure appropriate dissemination of findings to broad audiences [23–25], and contribute to the development of more effective interventions [24–28]. Our long-standing CBPR partnership in North Carolina (NC), comprised of Latine community members, representatives from Latine community organizations, health care providers, and academic researchers [6, 29], led this qualitative research, including developing and revising interview questions, ensuring recruitment of diverse participants to reduce potential bias, co-leading focus groups, and analyzing data. Specifically, in collaboration with grassroots community organizations, our CBPR partnership ensured the recruitment of diverse participants to reduce potential bias by seeking representation from various groups, including individuals residing in different cities, of different ages, genders, of various countries of origin, and diverse occupations, including farmworkers.

### Setting and participants

We recruited participants from locations across NC. NC is home to more than 1 million mostly immigrant Latine persons, many of whom speak Spanish primarily [30]. The COVID-19 experiences of the Latine population in NC may reflect experiences nationally [31, 32]. Using our extensive CBPR partnership network, in summer (May-August) 2022, we recruited and conducted in-depth interviews with 30 representatives from health and service organizations and 7 focus groups with 64 diverse adult Spanish-speaking Latine persons.

### Data collection

Interview and focus group guides assessed information and perspectives related to COVID-19 testing, vaccination, and booster uptake, separately, across the social ecological model (individual, interpersonal, institutional, community, and policy) [33]. Interviews also assessed experiences with program and service delivery. Interviews were conducted virtually in English (n = 24) or Spanish (n = 6). All focus groups were conducted in Spanish by native (Guatemalan and Peruvian) and/or bilingual Spanish speakers, in person (n = 3) or virtually (n = 4). All participants gave informed consent, provided information to allow team to identify them during data collection, and received $50 compensation. Demographic data were collected from each participant; focus group participants were provided an additional $30 for demographic questionnaire completion.

Human subject oversight was provided by Wake Forest University School of Medicine Institutional Review Board and the study is registered with ClinicalTrials.gov (NCT05302908). All participants were provided with written study information sheet and gave verbal informed consent.

### Data analysis

Interviews and focus groups were recorded and transcribed. Constant comparison, an approach to grounded theory, was used to analyze data. Constant comparison combines qualitative coding with simultaneous comparison; initial observations are refined throughout data analysis [34]. Because of the formative nature of this study, we aimed to identify the breadth of experiences, not to quantify them. A group of four analysts (including one community partner) coded transcripts and developed matrices to identify similarities and differences within and across groups (interviews and focus groups) and develop themes and domains. Discrepancies were resolved via discussion.

## Results

### Participants

Table 1 summarizes participant characteristics. Most interview participants self-identified their ethnicity as Latine (79%), their race as White (46%), and most had at least a college degree (67%); with an average age of 49 years old (*SD* = 12) and diverse organizational roles. The focus group participants were all Latine, representing Mexico and countries across South and Central America, and most had a high school degree or equivalent (79%); with an average age of 40.5 (*SD* = 14).

**Table 1:** Demographic characteristics of focus group and interview participants.

Characteristic	Focus groups* n (%) or mean ± SD	Interviews* n (%) or mean ± SD
Ethnicity		
Latine	62 (100)	19 (79)
Other	0	4 (17)
Missing	0	1 (4)
Hispanic/Latino/Spanish origin		
Mexican, Mexican American, Chicano	27 (44)	8 (42)
Puerto Rican	2 (3)	2 (11)
Colombian	10(16)	1(5)
Another Hispanic, Latino, or Spanish origin	20 (32)	7 (37)
Salvadoran	3 (5)	1 (5)
Dominican	0	0
Cuban	0	0
Prefer not to answer/Missing	0	5 (21)
Race		
Black or African American	0	1 (4)
White	2 (3)	11 (46)
Some other race	44 (71)	9 (38)
Multiracial/Multiple races	1 (2)	1 (4)
Prefer not to answer	15 (24)	2 (8)
Age (years)	40.5 ± 13.6	49.0 ± 11.6
Missing	5 (8)	2 (8)
Gender identity		
Man	18 (29)	4 (17)
Woman	40 (65)	19 (79)
Transgender woman/Male-to-female (MTF)	4 (6)	0
Missing	0	1 (4)
Education level		
Less than high school	13 (21)	0
High school graduate/GED/Some college	44 (71)	6 (25)
College degree or higher	5 (8)	16 (67)
Prefer not to answer	0	2 (8)
COVID-19 vaccination (at least one)	55 (89)	21 (88)
Organizational role		
Latine-serving organization representative		13 (43)
Community health worker		7 (23)
Clinical staff		4 (13)
Health care provider		3 (10)
Health department or state government staff		3 (10)

*Note. Demographic data provided from 62 of 64 (97%) focus group participants and 24 of 30 (80%) of interview participants (with the exception of organizational role, n = 30).

### Themes

Twenty themes emerged organized into four domains related to COVID-19 testing, vaccination, and booster uptake (summarized in Table 2): (1) general perceptions; (2) barriers; (3) facilitators; and (4) strategies and recommendations for promotion.

**Table 2:** Domains and themes from interviews and focus groups related to COVID-19 testing, vaccination, and booster uptake.

**General perceptions about COVID-19**
COVID’s disproportionate impact on Latine communities led community members to take it seriously at the onset of the pandemic (focus groups)
The perceived seriousness of COVID-19 has decreased over time among Latine communities (focus groups)
Latine communities distrust, are discouraged from using, and have difficulty accessing and navigating the US health care system (focus groups and interviews)
**Barriers to testing, vaccination, and booster uptake**
Misinformation and lack of information about COVID-19, testing, and vaccines (e.g., social media perpetuating myths and conspiracy theories) (focus groups and interviews)
Religious factors (e.g., some churches speaking against getting the vaccine) (focus groups and interviews)
Language-related barriers (e.g., home test kit instructions) (focus groups and interviews)
Testing and vaccination staffing (e.g., discrimination, language services) (focus groups and interviews)
Testing and vaccination logistics (e.g., documentation needs, long lines, hours) (focus groups and interviews)
Implications of positive test results (e.g., job security, quarantine options) (focus groups and interviews)
Side effects of vaccination (e.g., personal or by a known peer) (focus groups and interviews)
Gaps in knowledge related to need, eligibility, and access for boosters (e.g., changing availability by age and/or for children) (focus groups and interviews)
**Facilitators of testing, vaccination, and booster uptake**
Increasing comfort with testing process (e.g., home tests) and vaccination (e.g., increased communication about vaccine safety and efficacy) (focus groups and interviews)
Service provision by Spanish speaking staff in familiar locations (e.g., mobile clinics, Latine events) (focus groups and interviews)
External requirements for vaccination (e.g., job, social events, and travel) (focus groups and interviews)
Peer and community support and/or others’ positive experiences with testing and vaccination (focus groups and interviews)
Motivation from desire to avoid getting critically ill and/or hospitalized (focus groups and interviews)
**Strategies and recommendations to promote testing, vaccination, and booster uptake**
Testing and vaccination programs that address barriers (e.g., use of familiar spaces, extended program hours, walk-in and online appointments) (focus groups and interviews)
Culturally congruent education, communication, and service provision (e.g., community leaders, media, and call centers) (focus groups and interviews)
Utilizing personal stories of positive testing and vaccination experiences and familiar communication outlets (e.g., community social networks, WhatsApp groups, going door to door in high Latine census communities, and Spanish-speaking television stations) (focus groups and interviews)
Use of community health workers and peer navigators to address specific barriers (e.g., education around community immunity and booster eligibility) (focus groups and interviews)

#### General perceptions about COVID-19

Focus group participants reported that Latine communities were hard hit by COVID-19, thus took it seriously from the onset of the pandemic. Many participants knew people (family members or friends) who had died or experienced serious illness from COVID-19. Though there was general recognition that COVID-19 was less threatening over time, some (e.g., farmworkers) still viewed it as a serious matter. As highlighted by a focus group participant:

Well, okay. I was saying that COVID is still a worrisome topic because, well, it hasn’t disappeared 100 percent. Right? It’s still–it’s latent. But in the community, it feels like it’s being managed with more tranquility or with more confidence, vaccines. (Focus Group (FG) 0728-NC statewide, virtual)

Focus group participants reported that many Latines do not trust, are discouraged from using, and have difficulty accessing and navigating the U.S. healthcare system, especially for non-English speakers and for those who are accustomed to healthcare systems in other countries. This was reported in a focus group:

Why the distrust? Because–because as Hispanics, that’s how our culture is. It’s simply–we’re from countries where there are no public services and public services are very deteriorated. And here, when–and we come with that mentality of, “I’ll go to the doctor only when I feel sick, not when I’m okay. I won’t go to a routine exam, because I don’t need it, because I’m not sick. So, our mentality and our culture teaches us that…Our culture teaches us not to trust, it teaches us not to go to the doctor, it teaches us to not accept things because before, in the past, they were being used to do medical or clinical studies to create medications–certain people, communities were used in certain countries. So, all of that shot off here in the United States, and let’s not forget about our countries, where there was also chaos. (FG 0615-Forsyth County, NC, in-person)

Notably, participants reported that Latine communities fear using public health services given experiences with racial/ethnic discrimination, anti-immigration rhetoric, and policies and laws that discourage or restrict access. Participants reported that publicity around policies, such as Section 287(g) of the Immigration and Nationality Act and the Secure Communities program,(35) sanctuary city status,(36) and “public charge” (term used by U.S. immigration officials and the media for individuals considered likely to become dependent on the government for subsistence(37)), reduced perceived access to and discouraged use of COVID-related services, as noted:

I don’t know people who found it difficult, but I know many people, especially Latinos, who don’t know that they can get home tests at home. I don’t know if this is because they think or are afraid that they’ll be denied because of their status, some of them who don’t have documents. And they think they can’t get them. And that’s one of the questions that many people have who think they can’t get them because they’re undocumented. And I don’t know if that’s an obstacle for them. (FG 0623-NC statewide, virtual)

Participants also reported many Latines hold concerns that seeking services might expose them/family members to immigration enforcement actions or negative legal consequences related to immigration status.

#### Barriers to testing, vaccination, and booster uptake

Many barriers to COVID-19 testing, vaccination, and booster uptake were reported. Participants shared that many Latines distrusted the information received during the onset of the pandemic (especially since little was available in Spanish). This contributed to the circulation of misinformation and myths about COVID-19. Some participants reported hearing that COVID-19 did not exist and was an invention to control and confine people, or that the virus was created intentionally to make people sick and then sell them vaccines. Participants reported various forms of vaccine-related misinformation (e.g., fertility issues and microchips) from several sources, including friends, religious leaders, and media. Media, both social and traditional forms, fueled misinformation; one provider stated that many Latines, particularly young people, were getting information from social media that the vaccine was “dangerous”, not tested, or did not prevent COVID-19. This was discussed frequently, including in a focus group:

I watched a lot of TV and heard a lot on social networks that vaccines were a chip that was implanted in you, that it was part of a government strategy to keep you under control, mainly to keep you under control. So that was my fear. Many comments were made and they related it to biblical things, which was the mark of the devil, many things that happened. (FG 0623- NC statewide, virtual)

Participants also reported that initially there was not enough known about vaccines which contributed to doubts about safety and efficacy.

Participants reported that religious institutions were an important source of COVID-19-related information. While some churches supported and promoted protective measures, others had leaders who viewed COVID-19 prevention methods as a threat to religious practice through restricting congregational meetings and temporarily closing church facilities. This underscored the myths about how COVID-19 is a lie intended to distance people from churches/God. Both focus group and interview participants stated that some churches suggested COVID-19 is a sign of the end of the world and thus people should not accept preventive measures because God will protect them, as highlighted:

We saw so many things, I’m sorry to say it, but at the church level, they–they caused a terrible scandal, like, “They’re shutting down our churches. They don’t want us to gather. That’s all a lie.” I believe in God and this is what a pastor told me, “Sister, if you believe in God, nothing–this is a lie. None of this will happen. God will protect you. The blood of Christ will protect you.” There’s a–there was a–an imminent and latent emergency in the community and people continued to not believe it due to our culture being the way it is. (FG 0615-Forsyth County, NC, virtual)

Similarly, an interview participant reported:

There were very few pastors who heeded the plea. Because they thought, “This is a lie. What they want is to remove us from gathering in churches. What they want is–” One of them–I heard so many comments. One of them said, “This is from the devil because what they want is to prevent us from continuing to adore our Lord and serving him the way we do.” Another one said, “Sister, get–do you believe in God? Get on your knees and ask for his forgiveness because this is an exaggeration. This will pass. God has revealed it to me and nothing is going to happen.” Others said, “This shall pass with fasting and prayer. We all have to fast together. And–and that’s what we have to do.” There were so many of them stating this in the churches and then the Hispanic community would say, “No, what you guys are telling us is a lie. We’re not going to believe or accept it. (Interview (I) 0705-Winston-Salem, NC, woman, virtual)

While participants reported that most Latine community members hold more science-based views, these rumors are part of the general context that situates attitudes about and creates barriers to COVID-19 testing, vaccination, and booster uptake.

Participants reported that language-related barriers affect testing, vaccination, and booster uptake. Participants noted that home test kit instructions did not always include Spanish-language instructions, as reported by an interview participant, “At first home tests didn’t have Spanish instructions, so we created a video on social that was a hit” (I 0512-Raleigh, NC, woman, virtual). In addition, testing and vaccination sites without Spanish-speaking staff or Spanish-language information were described as missed opportunities to provide culturally congruent services by participants:

I saw many people in the pharmacy who don’t speak English. Many of them didn’t have a car. Many didn’t know where to go. It’s very easy because many people–the answer to know which website to go to, how to fill out the application, because in [urgent care clinic] you have to put your name, why you go there, if you have insurance. And it costs them a lot of work if it’s in English. Many times I had to translate and help them fill out the forms online, send them to the address and help them get there. I think someone who doesn’t speak English is going to have a hard time knowing where to go. (FG 0707-Forsyth County, NC, in-person)

Latines living in urban areas reported more access than those living in rural areas, including farmworkers, to Spanish-language information and health services.

Focus group participants noted other staffing challenges, including perceived discrimination. Participants shared being offered one option for the type of vaccine they could receive (i.e., the one dose Johnson and Johnson vaccine) while perceiving that White clients were offered multiple types of vaccine in the same location. The focus group comprised of farmworkers discussed this in depth:

In the city where we live is a farming town. The closest city is [city]. Only Americans live in that city, retired gringos, in [city]. And that city had all the resources, all the tests. For example, there you had to go to the closest cities to take the test. And the agricultural towns were the last ones, why, because we’re immigrants, because we’re agricultural, and we don’t matter so much. We don’t have social security. No. I think there’s that. And they didn’t test you in [city] if you weren’t from [city]. If you came from [town] to [city] to take the test, they said no because it’s not your county. It had to be the people of [city] first. (FG 0827-Hendersonville, NC, farmworkers, in-person)

Based on these negative experiences, participants were less willing to return for additional vaccine doses/boosters. This included how experiences with the immigration process influenced accessing health services, as highlighted:

There is a lot of mistrust, misinformation, tied to how people are treated while immigrating…they just got stuck in an arm at some detention facility, don’t know what it was. (I 0510-Wilmington, NC, woman, virtual)

Other logistics served as barriers. Participants reported confusion among Latine communities about whether identification or other documentation were required for testing or vaccination/boosters. Participants described how early in the pandemic Latine communities experienced substantial difficulties accessing sites that provided timely and affordable testing. Due to work/school schedules and location as well as technological and transportation issues, participants reported that services remain difficult to access during standard business hours.

I mentioned it to someone working in the Union municipality and I said, ‘This is happening. Have you been concerned about the people in the fields?’ Which is the same part of the county. They’re in the North Carolina zone, but those people are left over there. I mean, I took it upon myself to go see what was going on. And I would ask them. Because they were Latinos. ‘Hey, sir, have you gotten the vaccine? Have you gotten tested?’ ‘Oh, no.’ Because they’re camps. Now, what happened? That [name] County can’t go into those camps because they’re private property. But there’s a reality that’s very…that scared me, because they’re still working for North Carolina agriculture. They’re located there in the camps, but it’s like they don’t count as normal civic life. I mean, that’s what I experienced and it pained me so much. Because I think, people outside of the camp had access to take their truck, their bus and go get tested or get the vaccine and those people couldn’t. (FG 0714-Mecklenburg & Union Counties, NC, virtual)

Barriers were further highlighted in interviews:

The use of technological means is still a problem. In her organization, people can call and help you make appointments. But many people cannot use technological means. That is why health promoters are important because they assist people in everything they need and give them a follow-up. (I 0706-Durham/Carrboro, NC, woman, virtual)

The implications of a positive test result are barriers for testing. Overall, Latines perceive tests as a reliable means of assessing COVID-19 infection, some were unsure of the procedures were required (e.g., isolation) with a positive result and were concerned about missing work. While some focus group participants reported getting tested as part of requirements to go back to work/school, others stressed that many Latines have jobs without sick leave protections or where they feel that the employer would hold COVID-related absences against them.

The community is not interested in getting tested because they cannot go to work if the test results are positive. The test doesn’t help them get paid for those days of isolation in their jobs. They cannot afford to miss work and not get paid; they must keep working to feed their family. Many workplaces tell them that even with COVID, they come to work if they don’t have symptoms. All the sectors where undocumented people work in the same situation; they don’t offer them any benefits. They don’t get paid if they don’t show up to work. Also, Latinos can’t isolate themselves because they have to go out in person to do their shopping; they don’t have access to a credit card because they don’t have social security, so they don’t have Amazon, nor do they have access to make purchases online, they go to the store directly. (I 0720-Raleigh, NC, woman, virtual)

Similarly, a focus group participant stated:

Our community doesn’t have health insurance. And our community is–the majority are undocumented, so they work–if they work one hour, that’s the hours’ worth of pay they’ll be getting. So, they didn’t want to stop working. Another thing was that employers didn’t want to let their employees go get tested, much less let them go, because they’d lose their workforce, and their businesses would go down. (FG 0615-Forsyth County, NC, virtual)

Further, the living situation of some Latine families did not easily accommodate recommended isolation practices and some participants described a lack of awareness of sources of, or eligibility for, aid that were available to address these concerns, as discussed:

Previously at my job you needed a negative test to return to work. If you’ve taken the test, they must know the day you did it, and you have to send the result when you have it. Many people don’t get tested and they don’t want to know because they need money for food, rent, and anything else. (FG 0623-Statewide, NC, virtual)

Participants also worried about side effects. Some focus group participants reported that they (or someone they knew) had a negative reaction after their initial vaccine and did not get further doses/boosters:

One person who was vaccinated and one who was not, if they both got sick at the same time, in my experience, the person who had been vaccinated was sick for more days and had more fever and more time in bed. And the person who wasn’t vaccinated was better in a few days, maybe with a little fever. But I also have a family member who had the first vaccine and was in the hospital, got infected with COVID and was in the hospital for seven days even though he was vaccinated. He only got one vaccine because he got COVID and he didn’t want the other one anymore. (FG 0707, Forsyth County, NC, in-person)

Side effect concerns resulted in some parents not vaccinating their children, even if they themselves had received the vaccine and experienced no side effects as reported by an interview participant:

Sometimes adults get vaccinated but don’t want to vaccinate their children. They are worried about side effects on their children; some rumors that the vaccines will leave them with infertility. (I 0808-Siler City, NC, woman, virtual)

Knowledge was low about availability of and eligibility for boosters (e.g., changing availability by age). Many participants questioned the need for boosters (e.g., frequency and different intervals from flu shot). As such, participants reported lower willingness to receive boosters when made available, as noted:

Not all people are putting on boosters. Many people think they are no longer necessary because they have already gotten sick with COVID-19. (I 0719-Greenville, NC, woman, virtual)

#### Facilitators of testing, vaccination, and booster uptake

Participants reported increased comfort with testing and vaccination procedures among Latine communities:

Many people–for example, home test… The same thing happened with my daughter. She watched the nurse practitioner how she did it, she just left it at that. And she said, ‘It’s not painful.’ And she said, ‘It’s not if we do it carefully.’… If we teach how to do it, maybe people will lose that fear of what they’re going to put in my nose and whatever. (FG 0623-NC statewide, virtual)

The availability of home testing as well increased education and communication about vaccine safety and efficacy in supportive clinical environments were factors identified as enhancing access and uptake. This was noted in a focus group:

And we said, we must have our family safe, and if they’re telling us–because we investigated in several clinics listening–this is safe. I didn’t hear that they were vaccinated and died, at least not around me. (FG 0827-Hendersonville, NC, farmworkers, in-person)

Participants commented that the availability of Spanish-language information was greatly improved from the onset of the pandemic. This was highlighted in one focus group:

We along with several groups were working in that part and we provided documentation in Spanish, because the state didn’t have anything in Spanish yet. So, in order to explain to the families we also, for example, comics to make it easier for families to understand about Covid and all that and we took things through organizations they trust. (FG 0714-Mecklenburg & Union Counties, NC, virtual)

Participants also reported increased ease when receiving services from personnel who spoke Spanish or from known and trusted institutions, such as churches or clinics, as reported:

We have long term history at our clinic, so Spanish speaking families trust the place. We are working to be proactive. We called patients, didn’t wait for them to come to clinic. And if kids come into clinic, then the whole family could get vaccinated in the pediatric clinic. (I 0713-Winston-Salem, NC, woman, virtual)

External requirements were a vaccination facilitator. As one interview participant commented: “For people to get vaccinated and tested, they should make it mandatory. Vaccines and tests should be required in work and study places: (I 0621-Greensboro, NC, man, virtual). When vaccines were initially available, employers’ requirements were a strong motivator. Notably, these experiences differed by participants’ immigration status (e.g., legal permanent residents vs. temporary workers). Farm workers reported feeling more isolated and more beholden to their employers, so the choices of the employer (obligating them to get the vaccine or not providing them opportunities to get vaccinated) often dictated their behaviors. More recently, social event and travel requirements motivated individuals to get vaccinated; participants reported they wanted to get back to “normal” and thus motivated to get vaccinated. This was discussed frequently, including:

First of all, because they would say it at work. My idea was to work, but well, no… I need to have the vaccine and second of all, because we were going to travel and they were requesting it at the airport, at restaurants. (FG 0726-Mecklenburg County, in-person)

Participants emphasized the crucial role of peer influence and support. For many Latines, if someone they knew had been vaccinated and had a positive experience, they were more likely to seek vaccination. This was especially true if a community leader had been vaccinated, highlighting the important role of informal community leaders. The important role of peer influence was described:

But if you’re someone who is scared and you do want to get vaccinated, I’d say, ‘Well, let’s go. I’ll go with you so you feel comfortable and so you don’t feel alone.’ So, ‘I’ll keep following up with you. Let’s go to the next appointment.’… For example, my own family didn’t want to get vaccinated, so I had to say, ‘Look, nothing happened to me. I’m still here. I didn’t transform,’ because, well, there was that topic of the zombies. So–[laughter] yeah, that part. (FG 0728-NC statewide, virtual)

Another facilitating factor included worry about becoming seriously ill or hospitalized. Latines with personal experiences with COVID-related morbidity/mortality were especially motivated to engage in prevention behaviors, such as:

I did know a person who had Covid and was hospitalized for about six months. Then he had other complications because he was already an old person. And he was left with many sequelae. He was left with after-effects. And many people have sequelae after Covid. That’s why it’s better to get vaccinated. (FG 0726-Mecklenburg County, in-person)

This resulted in one interview participant suggesting, “One strategy is sharing stories of families affected by COVID, having conversations about the importance of vaccination” (I 0613-Charlotte, NC, woman, virtual). In addition, some who had initially avoided vaccination got the vaccine after they had been sick with COVID-19 to avoid future illness.

#### Strategies and recommendations

Participants described existing strategies and additional recommendations to support testing, vaccination, and booster uptake. Some strategies highlighted included use of familiar locations and spaces for the provision of services, as recommended:

Another thing is also that families are finding the vaccine through places they know or at churches or in different places they trust, so they are no longer afraid of that. (FG 0714-Mecklenburg & Union Counties, virtual)

Additional strategies included targeted mobile testing, extended hours, and walk-in appointments (*and* online appointment services) to address transportation, schedule, and technology-related concerns. The direct distribution of home tests attenuated many immigration-related concerns, and thus home testing is the preferred method.

Participants underscored the importance of culturally congruent education, communication, and service provision that addressed importance of testing, vaccination, and boosters in Spanish-by-Spanish speakers. For instance, one focus group discussed:

I would say that the information about the pandemic, people were getting it from–from social media. Right? From Facebook, from, I don’t know, from YouTube. I think some people…I received several videos, right? About the pandemic. So, social media, YouTube, and of course, information from friends and family. I think those are–you could summarize it that way, those were the three sources, basically. And I think a fourth one would be Univision and–if you only speak Spanish here, I think Univision was a very, very important source of information about the pandemic. …There were sectors within the pastors and within the churches that were in complete disagreement with all these [anti-vaccination] views. And they would encourage their congregants to get vaccinated, right? And a lot of pastors even went as far as letting their churches be used for vaccination events. (FG 0615-Forsyth County, virtual)

Participants also described the utility of sharing of personal stories of positive testing and vaccination experiences by other Spanish-speaking Latines. Communication avenues that were identified as useful included community leaders (e.g., supportive religious leaders), social media (e.g., moderated community WhatsApp and Facebook groups), Spanish-language media and services (e.g., Univision and call centers), and going door-to-door in high Latine census communities. For instance, one interview participants discussed the benefits of their group, “We utilize a very active WhatsApp group with community and neighborhood leaders, focused on six specific zip codes, to share COVID information and resources” (I 0607-Charlotte, NC, woman, virtual).

Given that Latine communities do not always trust government systems, participants reported that it was crucial to support community organizational capacity to improve service access, as reported:

That’s what we have to keep doing, try to keep educating our communities and keep giving them the right information… We’re here with you receiving information, and this same information we can share with people in the community outside to let them know the resources that exist for our communities. (FG 0623-NC statewide, virtual)

Participants highlighted the integration of community-based peer health navigators (who use natural helping) and more formally trained CHWs. Spanish-speaking Latine peer navigators and CHWs, connected to local clinics, organizations, and health departments, were identified as crucial in COVID-19 testing, vaccination, and booster uptake, as noted:

I recommend hiring health promoters, as other counties are doing…these promoters should be someone of Hispanic origin who comes out of their midst because they know the community. They could be students from the health area. The community’s leaders must be people with whom the community identifies and feels trusting. (I 0705-Winston-Salem, NC, woman, virtual)

CHWs devoted substantial efforts to educate Spanish-speaking individuals (e.g., community immunity and booster eligibility) and directly address misinformation. They also encouraged testing and vaccination sites to attend to factors that function as deterrents. For instance, they offered feedback to limit required information to the minimum necessary for public health reporting (e.g., name, address, and birthdate). CHWs described the development of a “know your rights” card for Spanish speakers specifying which types of identification were required for services and created Spanish-language videos about administering home testing and what to expect at testing and vaccination sites. To address immigration enforcement concerns, CHWs worked with local law enforcement to minimize presence at community sites.

## Discussion

This study provided critical insights into factors affecting COVID-19 testing, vaccination, and booster uptake among Spanish-speaking Latines, a group disproportionately affected by COVID-19 [2, 5–7]. According to participants, limited access to health services continues to affect Latine communities, and the pandemic highlighted the ways that sociocultural determinants affect use of services to diagnose and prevent COVID-19.

We identified general and culturally specific barriers to testing, vaccination, and booster uptake at multiple levels; both need to be considered in the development of tailored interventions for Spanish speakers. Individual-level factors included lack of knowledge around COVID-19 prevention strategies and fears related to immigration documentation status and accessing services. Interpersonal factors included low trust in healthcare providers, COVID-19 myths (e.g., from social media and some religious leaders), and lack of access to culturally congruent information (e.g., recommended quarantine/isolation protocols and vaccination safety and efficacy). Institutional factors included challenges with testing and vaccination site logistics and staffing (e.g., lack of bilingual staff and distrust of government systems). Community factors included limited transportation options. Finally, policy factors included the implications of a positive test and vaccination/booster side effects (e.g., missing work); on the other hand, external requirements, including from employers, could be a facilitating factor for vaccination and boosters.

Our findings confirm the barriers identified in prior research [19, 21, 22]. Despite barriers, we heard largely encouraging reports about the increasing receptivity to testing, vaccination, and boosters in this community. Community-based organizations have devoted substantial efforts to enhancing access to support testing, vaccination, and booster uptake among Spanish-speaking Latines. This has included information provision and service delivery in Spanish-by-Spanish speakers through culturally congruent strategies, like offering services in familiar spaces, partnering with supportive religious leaders, disseminating information through social media, and communicating positive peer experiences. Both CHWs and peer navigators, especially those that speak Spanish, play an important role in providing accurate and culturally congruent information to address barriers to service access and communicate information.

### Limitations

These data should be situated within the study context. Data were collected in NC using convenience sampling; NC has a large Latine population and the demographics are similar to Latine populations in other U.S. states [35–38]. Our participants represented a variety of organizations and Latine communities (e.g., farmworkers) from rural and urban locations which further ensures the identification of the sociocultural determinants of service uptake in this community. However, individuals agreeing to participate may have different experiences. These data were collected during a time of diminished sense of seriousness related to COVID-19 due to reductions in community-level restrictions (e.g., school closures and mask mandates) as well lower rates of serious illness. Data were collected prior to the release of the bivalent booster (September 2022) which may affect awareness, access, and uptake.

### New contributions

Based on this formative work, our CBPR partnership is developing *Nuestra Comunidad Saludable* (*Our Healthy Community*) [33], an intervention to increase ongoing testing, vaccination, and booster uptake among Spanish-speaking Latines. The intervention will harness the strengths of community-based peer navigators who will serve as educators and advocates within their social networks. Such an approach is less resource-intense than some CWH strategies and is effective in addressing health disparities in underserved communities through intervening on multilevel factors [28, 29, 39–41]. Effective peer navigators are part of the networks in which they work (e.g., self-identity and socioeconomic status); understand community needs; communicate in the language of members; and incorporate culture to promote health [29, 39–45]. As our partnership has learned, peer navigation strategies can be sustainable as they often continue their work after a study ends [46, 47].

In *Nuestra Comunidad Saludable*, we will test whether trained peer navigators can increase COVID-19 testing, vaccination, and booster uptake among Spanish-speaking Latines through blending in-person interactions and mHealth (mobile health) strategies using social media [33]. Peer navigators can reach those who are considered “hard-to-reach” and peer navigation interventions can be targeted, tailored, and personalized [48]. Navigators will *target* Spanish-speaking Latines in need of testing, vaccination, and/or boosters; *tailor* their interactions to the strategies preferred for each participant; and *personalize* activities/messaging to the priorities and needs of each participant [49].

As the pandemic continues to evolve, prevention efforts for all communities must evolve as well [50]. A need exists to understand and intervene upon factors associated with diagnosis and prevention among Latine communities. Our results underscore important sociocultural determinants of testing, vaccination, and booster uptake to consider ensuring culturally congruent strategies and support health equity for Spanish-speaking Latines in the United States.
